# ﻿A revision of the genus *Trichohoplorana* Breuning, 1961 (Arthropoda, Insecta, Coleoptera, Cerambycidae, Lamiinae, Acanthocinini)

**DOI:** 10.3897/zookeys.1160.103596

**Published:** 2023-05-10

**Authors:** Gui-Qiang Huang, Dong-Shuo Liu, Rong-Chuan Xiong

**Affiliations:** 1 School of Biological Science and Technology, Liupanshui Normal University, Liupanshui 553004, Guizhou, China Liupanshui Normal University Liupanshui China; 2 Unaffiliated, 401, Unit 1, Building 11, Xiying Qianjie, Tongzhou District, 101149, Beijing, China Unaffiliated Beijing China

**Keywords:** Hind wings, male terminalia, new faunistic records, new species, synonyms

## Abstract

A taxonomic revision of the genus *Trichohoplorana* Breuning, 1961 is presented. A junior synonym of *Trichohoplorana*, *Ipochiromima* Sama & Sudre, 2009, **syn. nov.**, is proposed. A junior synonym of *T.dureli* Breuning, 1961, *I.sikkimensis* (Breuning, 1982), **syn. nov.**, is proposed. *Trichohoplorana* is newly recorded from Vietnam. A new species, *T.nigeralba***sp. nov.** is described from Vietnam. *Trichohoploranaluteomaculata* Gouverneur, 2016 is newly recorded from China and Vietnam. Hind wings and male terminalia of *T.luteomaculata* are described for the first time. *Trichohoplorana* is redescribed, and a key to *Trichohoplorana* species is presented.

## ﻿Introduction

The genus *Trichohoplorana* was established by Breuning (1961) for *Trichohoploranadureli* Breuning, 1961. It presently consists of six species from Asia (Tavakilian and Chevillotte 2022). Mitono (1943) described *Acanthocinusshirakii* from China (Taiwan), then Gressitt (1951) transferred *A.shirakii* Mitono, 1943 to *Neacanista* Gressitt, 1940, and finally, Gouverneur (2016) transferred *N.shirakii* to *Trichohoplorana* and described *T.luteomaculata* from Laos (Houa Phan). Holzschuh (1989, 1990, 2015) described *T.juglandis*, *T.mutica*, and *T.tenuipes*, respectively, from South Asia.

In this work, new synonyms, new faunistic records, and a new species are provided. Wings and male terminalia are described for the first time. Consequently, *Trichohoplorana* now consists of seven species from Asia. A redescription and a key to all species of *Trichohoplorana* are presented.

## ﻿Material and methods

Specimens examined are deposited in following institutions and private collections:

**CDSL** Collection Dong-Shuo Liu, Beijing, China

**CHS** Collection Carolus Holzschuh, Villach, Austria

**CWW** Collection Andreas Weigel, Wernburg, Germany

**CXG** Collection Xavier Gouverneur, Rennes, France

**LPSNU** School of Biological Science and Technology, Liupanshui Normal University, Liupanshui, Guizhou, China

**MNHN**Muséum national d’Histoire naturelle, Paris, France

**SYSU**The Museum of Biology, Sun Yat-sen University, Guangzhou, China

The methods of taking photographs for Figs 2C–G, 4B–F, 6A–E followed Huang et al. (2020), and methods of photographing Fig. 3A–J mainly followed Huang and Li (2019) but were taken with a E3ISPM21000KPA camera and ImageView software. The terminology of hind wings vein follows Švácha and Lawrence (2014). The terminology of male terminalia follows Ślipiński and Escalona (2013).

## ﻿Taxonomy

### ﻿ Trichohoplorana

Taxon classificationAnimaliaColeopteraCerambycidae

Breuning, 1961

4C0326AE-72DA-5357-AF85-3F67EA7348E4

Trichohoplorana Breuning, 1961: 548; Breuning 1963: 534; Breuning 1978: 49; Löbl and Smetana 2010: 213. Type species: Trichohoploranadureli Breuning, 1961, by original designation.
Trichhoplorana Breuning 1977: 115 (misspelling).
Ipochiromima Sama and Sudre 2009: 384 (replacement name for Mimipochira Breuning, 1982: 25); Löbl and Smetana 2010: 209. Type species: Mimipochirasikkimensis Breuning, 1982, by original designation. Syn. nov.

#### Redescription.

Head distinctly narrower than prothorax, frons with a narrow, median furrow extending from base of clypeus up to apical margin of pronotum; eyes coarsely faceted, lower lobes of eyes distinctly far away from each other and longer than genae; antennae slender, distinctly longer than body, scape strongly expanded before apex, pedicle distinctly longer than broad. Pronotum transverse, with a tubercle on each side, punctured, with a pair of subuliform tubercles at sides of middle; prosternal process broad, with a longitudinal depression in middle, procoxal cavities closed posteriorly. Scutellum linguiform. Elytra covered with black or brown spots and a series of black or brown spots along suture; disc elongate, distinctly broader than pronotum at base, gradually narrow from near apical third, punctured, with a pair of tubercles at base and near scutellum, with a pair of bumps behind the tubercles; humeral angles rounded and slightly processed forward. Mesocoxal cavities closed externally to mesepimera. Femora strongly clavate.

#### Diagnosis.

*Trichohoplorana* is very similar to *Neacanista* Gressitt, 1940 in having the pronotum with a tubercle at each side, with a pair of tubercles at the sides of the middle, the elytra with a pair of tubercles at the base and near the scutellum, with a pair of bumps behind the tubercles, and a strongly clavate femora. However, *Trichohoplorana* differs from *Neacanista* in having the antennal scape strongly expanded before the apex (gradually thickened before the apex in *Neacanista*) and the pedicle distinctly longer than broad (broader than long in *Neacanista*).

#### Distribution.

Bhutan, China, India, Laos, Nepal, Vietnam (**new country record**).

#### Remarks.

**Figure 1.**
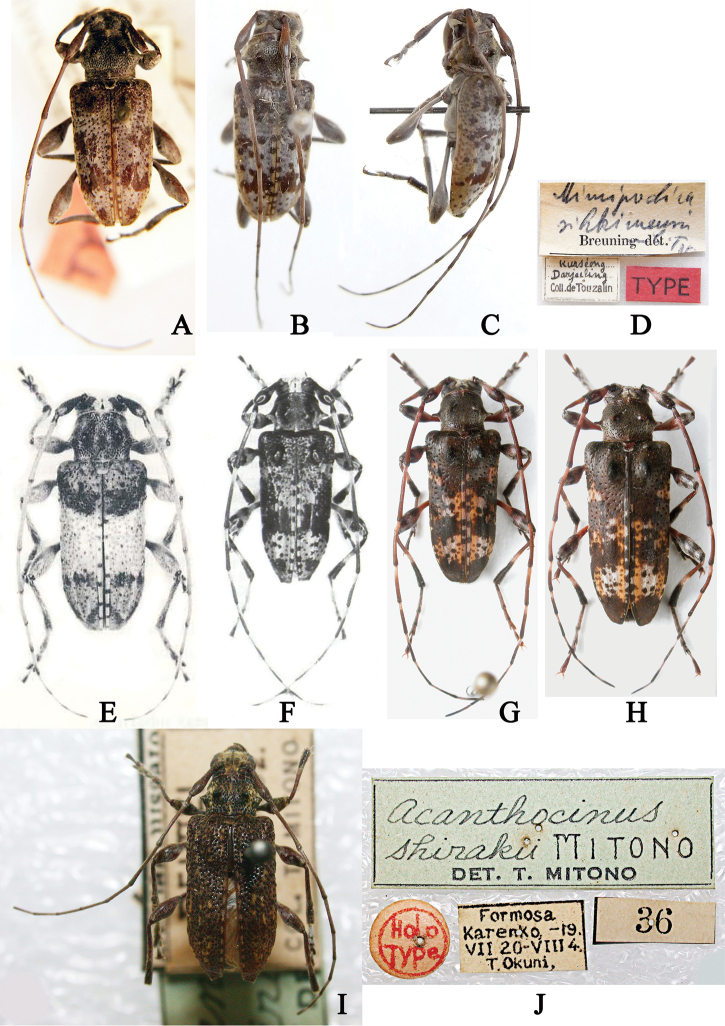
Types of *Trichohoplorana* spp. **A***Trichohoploranadureli*, holotype, male habitus, dorsal view (photo by Andreas Weigel) **B–D***Mimipochirasikkimensis***B** holotype, male habitus, dorsal view **C** holotype, male habitus, lateral view **D** labels (photos by Xavier Gouverneur) **E***Trichohoploranajuglandis*, holotype, male habitus, dorsal view (photo reproduced from Holzschuh 1989) **F***Trichohoploranamutica*, holotype, male habitus, dorsal view (photo reproduced from Holzschuh 1990) **G, H***Trichohoploranatenuipes***G** holotype, male habitus, dorsal view **H** paratype, female habitus, dorsal view (photos provided by Holzschuh) **I, J***Acanthocinusshirakii*: **I** holotype, male habitus, dorsal view **J** labels (photos reproduced from Huang et al. 2015).

Breuning (1982) established *Mimipochira* for *M.sikkimensis* Breuning, 1982, but this genus was a junior homonym of *Mimipochira* Breuning, 1956. Hence, Sama and Sudre (2009) introduced the new name *Ipochiromima*. After comparing photographs of the holotypes of *T.dureli* Breuning, 1961 (Fig. 1A) and *M.sikkimensis* (Fig. 1B, C), we consider these two species as belonging to the same genus, based on above redescribed characters. Thus, we treat *Ipochiromima* as a junior synonym of *Trichohoplorana*.

### ﻿ Trichohoplorana dureli

Taxon classificationAnimaliaColeopteraCerambycidae

Breuning, 1961

A5B1C2B3-A9B5-58C9-9130-0E0C99080B1F

Fig. 1A–D

Trichohoplorana dureli Breuning, 1961: 548 (type locality: “Pedong, Sikkim, India”); Breuning 1963: 534 (catalogue); Breuning 1978: 49 (redescription), pl. IV, fig. 15 (holotype); Löbl and Smetana 2010: 213 (catalogue).
Ostedes dureli : Breuning and Heyrovský 1961: 143.
Mimipochira sikkimensis Breuning, 1982: 26 (type locality: “Sikkim, India”). Syn. nov.
Ipochiromima sikkimensis : Sama and Sudre 2009: 384 (catalogue); Löbl and Smetana 2010: 209 (catalogue).

#### Type material examined.

*Trichohoploranadureli* Breuning, 1961: ***holotype***, ♂ (MNHN), Pedong, Sikkim, India, 1914, L. Durel leg., [examined from a photograph (Fig. 1A)]; *Mimipochirasikkimensis* Breuning, 1982: ***holotype***, ♂ (MNHN), Sikkim, India [examined from three photographs (Fig. 1B–D)].

#### Distribution.

Bhutan, India (Sikkim).

#### Remarks.

The differences between *T.dureli* and *I.sikkimensis* (Fig. 1A–C) mainly reflect in the shape of the elytral brown spots: the large spots near elytral middle, at apical third, and at apex. They are actually intraspecific differences; thus, we treat *I.sikkimensis* as a junior synonym of *T.dureli*.

### ﻿ Trichohoplorana juglandis

Taxon classificationAnimaliaColeopteraCerambycidae

Holzschuh, 1989

9A8D093A-253F-554A-A074-E34B2DF3A050

Fig. 1E

Trichohoplorana juglandis Holzschuh, 1989: 401 (type locality: “Menchunang, East Dochu-La, Thimphu district, West Bhutan”), fig. 8 (holotype, male); Löbl and Smetana 2010: 213 (catalogue); Weigel 2012: 408 (catalogue), pl. XXVII, fig. b; Lazarev 2019: 154 (catalogue).

#### Distribution.

Bhutan (Thimphu), India (Arunachal Pradesh).

### ﻿ Trichohoplorana mutica

Taxon classificationAnimaliaColeopteraCerambycidae

Holzschuh, 1990

DA36139F-5B35-5555-B67E-C9A4FB03B104

Fig. 1F

Trichohoplorana mutica Holzschuh, 1990: 193 (type locality: “Footpath from Sherpagaon to Ghora Tabela, Langtang Khola, Nawakot, C-Nepal”), fig. 11 (holotype, male); Weigel 2006: 506 (catalogue); Löbl and Smetana 2010: 213 (catalogue).

#### Distribution.

Nepal (Nawakot).

### ﻿ Trichohoplorana tenuipes

Taxon classificationAnimaliaColeopteraCerambycidae

Holzschuh, 2015

0EA86FF7-82F2-55A4-AC62-F3B6E1A4DD9C

Fig. 1G, H

Trichohoplorana tenuipes Holzschuh, 2015: 473 (type locality: “Bhalukhop, district of Taplejung, East Nepal”), figs 3 (holotype, male) and 4 (paratype, female).

#### Type material examined.

***Holotype***, ♂ (CHS), Bhalukhop, district of Taplejung, East Nepal, alt. 3000m, 11–21.V.2013, Emil Kučera leg., [examined from a photograph (Fig. 1G)]; ***paratype***, 1 ♀ (CHS), data same as holotype, [examined from a photograph (Fig. 1H)].

#### Distribution.

Nepal (Taplejung).

### ﻿ Trichohoplorana shirakii

Taxon classificationAnimaliaColeopteraCerambycidae

(Mitono, 1943)

DCE884F6-D2C8-5DD8-89F4-A967EB5F247E

Fig. 1I, J

Acanthocinus shirakii Mitono, 1943: 584 (type locality: “Reimei, Hassenzan, Taiwan”).
Neacanista shirakii : Gressitt 1951: 518 (catalogue); Breuning 1978: 40 (redescription); Hua 1982: 99 (catalogue); Nakamura, Makihara and Saito 1992: 95 (catalogue); Hua 2002: 218 (catalogue); Chou 2004: 326, fig (male); Hua et al. 2009: 95, pl. XCV, fig. 1091 (male and female); Löbl and Smetana 2010: 210 (catalogue); Huang et al. 2015: 560 (catalogue), figs 32 (holotype, male), 33 (holotype, labels), and 34 (4 in map).
Trichohoplorana shirakii : Gouverneur 2016: 72, figs 2a (holotype, male) and 2b (holotype, labels).

#### Distribution.

China (Taiwan).

### ﻿ Trichohoplorana luteomaculata

Taxon classificationAnimaliaColeopteraCerambycidae

Gouverneur, 2016

12D8D803-E07F-5286-871F-27F398D81D35

Figs 2 , 3 , 4 , 5

Trichohoplorana luteomaculata Gouverneur, 2016: 69 (type locality: “Ban Saleui, Massif du Mont Phou Pan, Houa Phan Province, Northeast Laos”), figs 1a, b (holotype, male) and 1c (paratype, female).

#### Type material examined.

**Figure 2.**
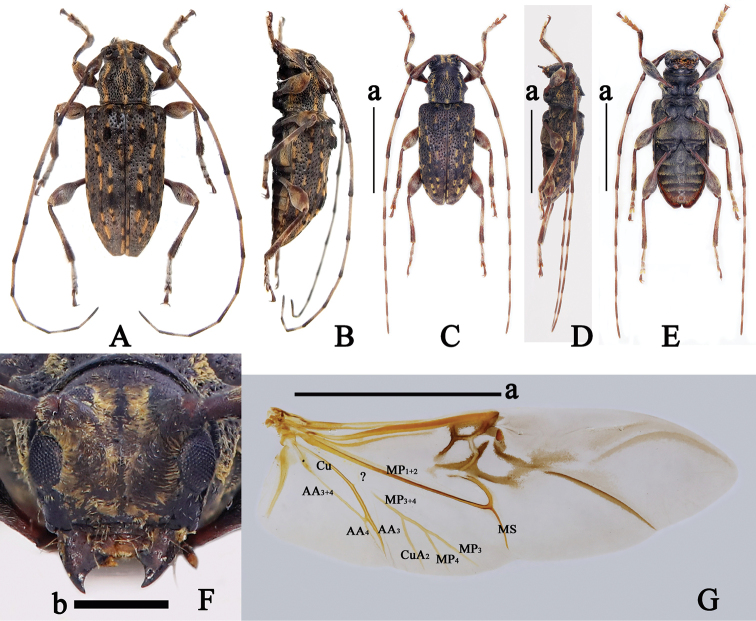
*Trichohoploranaluteomaculata*, males **A, B** holotype habitus **A** dorsal view **B** lateral view (photos by Xavier Gouverneur) **C–G** material from Hainan, China **C** habitus, dorsal view **D** habitus, lateral view **E** habitus, ventral view **F** head, frontal view **G** right hind wing, dorsal view. Abbreviations: A: anal, Cu: cubital, MP: medial posterior, MS: medial spur, ?: a vein of uncertain homology (either a crossvein or base of MP_3+4_ vein). Scale bars: 5 mm (**a**); 1 mm (**b**).

***Holotype***, ♂ (CXG), Ban Saleui, Massif du Mont Phou Pan, Houa Phan Province, Northeast Laos, alt. 1300–1900m, 1.V.2012, local collector leg., [examined from two photographs (Fig. 2A, B)]; ***paratype***, 1 ♀ (CXG), data same as holotype, but 2.V.2014 [examined from a photograph (Fig. 4A)].

**Figure 3.**
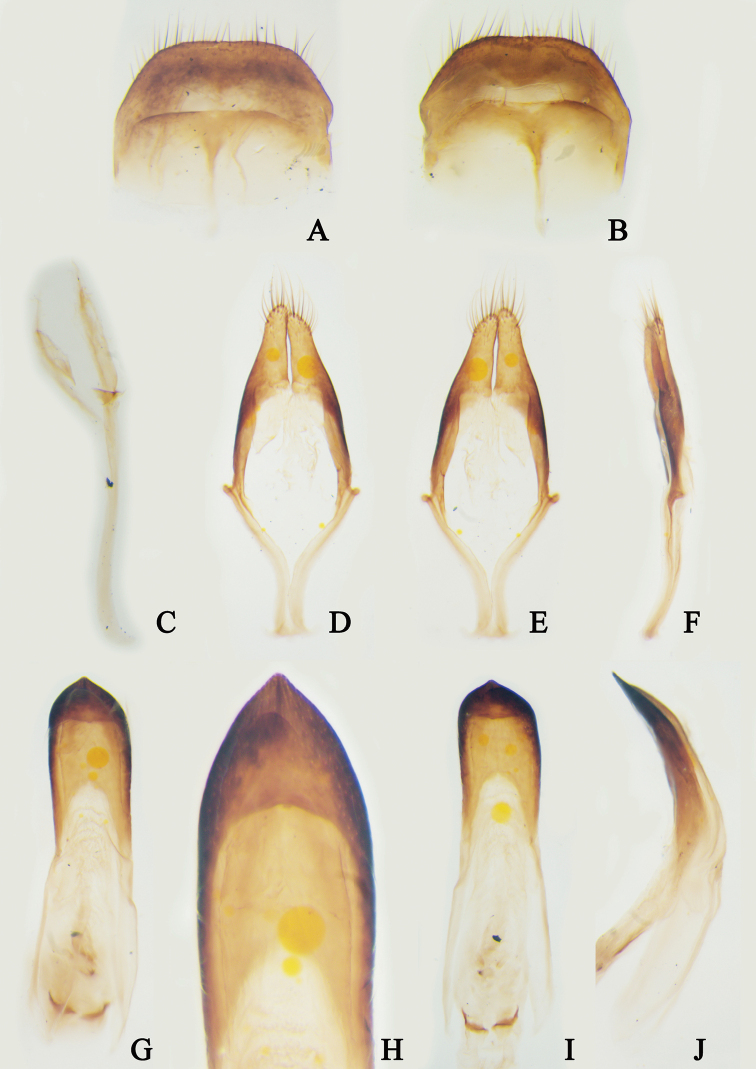
Male terminalia of *Trichohoploranaluteomaculata* from Hainan, China **A** tergite VIII, dorsal view **B** sternite VIII, verntral view **C** spiculum gastrale, dorsal view **D–F** tegmen **D** dorsal view **E** ventral view **F** lateral view **G–J** penis **G** dorsal view **H** dorsal view **I** ventral view **J** lateral view. Not to scale.

**Figure 4.**
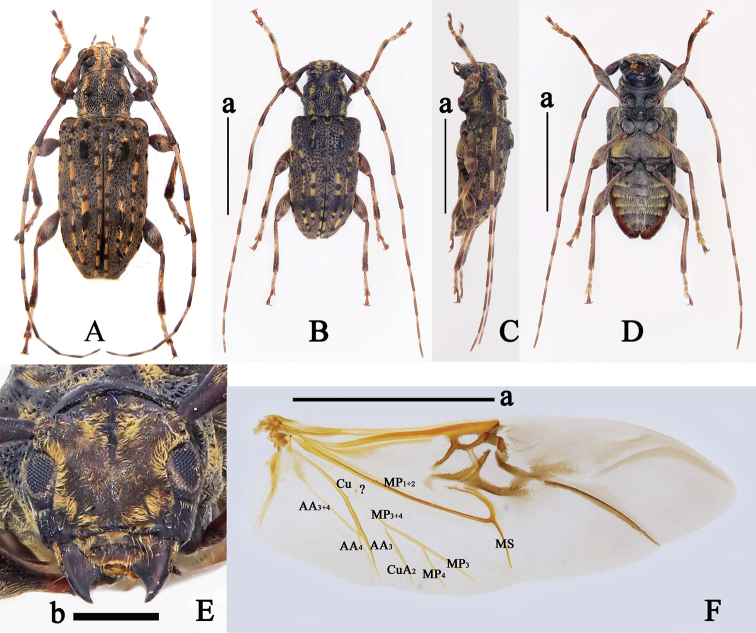
*Trichohoploranaluteomaculata*, females **A** paratype habitus, dorsal view (photo by Xavier Gouverneur) **B–F** material from Hainan, China **B** habitus, dorsal view **C** habitus, lateral view **D** habitus, ventral view **E** head, frontal view **F** right hind wing, dorsal view. Abbreviations: A: anal, Cu: cubital, MP: medial posterior, MS: medial spur, ?: a vein of uncertain homology (either a crossvein or base of MP_3+4_ vein). Scale bars: 5 mm (**a**), 1 mm (**b**).

#### Additional material examined.

**Figure 5.**
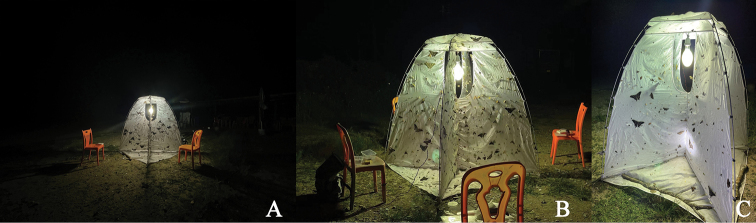
Scene showing collecting *Trichohoploranaluteomaculata* by light trap from Hainan, China (photos by Dong-Shuo Liu).

**China**: 1 ♀ (SYSU), Suoli village, Tongdao County, Huaihua City, Hunan Province, V.1981, Li-Jun Zhang leg.; 1♂1♀ (CDSL, figs 2C–G, 3, 4B–F), Jianfengling National Natural Reserve, Hainan Province, 7.VI.2021, 18°42'34.75"N, 108°52'34.50"E, alt. 954 m, Dong-Shuo Liu leg., collected by light trap (Fig. 5); **Vietnam**: 1 ♀ (LPSNU), Yen Bai Province, August 2020, local collector leg.; 1 ♀ (CWW), Gem. Ta Pin, Kreis Sapa, Lao Cai Province,22°22.196'N, 103°48.701'E, alt. 2318 m, 26.VI–01.VII.2017, N.H. Binh leg., [examined from a photograph provided by Andreas Weigel].

#### Supplementary description.

**Male** (Figs 2C–G, 3). Hind wings (Fig. 2G) with AA_3+4_ vein bifurcate near apical third; AA_4_ vein and AA_3_ vein closed to each other apically and not extending to margin; AA_3_ vein connected with Cu vein near apical third; CuA_2_ vein connected with MP_3+4_ vein near basal third of MP_3+4_ vein and not extending to margin; MP_3+4_ vein bifurcate near middle; MP_4_ vein, MP_3_ vein and MS vein not extending to margin; a short and vague uncertain vein (?, either a crossvein or base of MP_3+4_ vein) located between Cu vein and MP_1+2_ vein.

***Male terminalia*.** Tergite VIII (Fig. 3A) sparsely covered with short brown setae apically and at sides of apical third, nearly truncated at apex. Sternite VIII (Fig. 3B) anchor-shaped, sparsely covered with short brown setae at apical sides, apical margin slightly depressed; spiculum relictum distinctly longer than sternite VIII. Stem of spiculum gastrale more than 2.0 times as long as branches and curved towards dorsum at base (Fig. 3C). Parameres of tegmen sparsely covered with short brown setae on apical third and several long setae near apical fifth; each paramere gradually constricted from base to apex, but external margin slightly expanded near apex; apex of both parameres rounded and closed together; phallobase nearly 3.0 times as long as parameres and processed outward near middle; anterior tegminal strut curved outward (Fig. 3D–F). Penis curved towards venter, ventral plate distinctly longer and broader than dorsal plate and slightly sharp at apex; dorsal plate widely rounded at apex; apex of dorsal struts obliquely truncated (Fig. 3G–J).

**Female** (Fig. 4B–F). Hind wings (Fig. 4F) with AA_3+4_ vein bifurcate near apical third; AA_4_ vein and AA_3_ vein fused apically and not extending to margin; AA_3_ vein connected with Cu vein near apical third; CuA_2_ vein connected with MP_3+4_ vein near basal fifth of MP_3+4_ vein and not extending to margin; MP_3+4_ vein bifurcate near middle, MP_4_ vein, MP_3_ vein and MS vein not extending to margin; a short uncertain vein (?, either a crossvein or base of MP_3+4_ vein) located between Cu vein and MP_1+2_ vein.

#### Distribution.

China (Hainan, Hunan), Laos (Houa Phan), Vietnam (Lao Cai, Yen Bai).

### ﻿ Trichohoplorana nigeralba sp. nov.

Taxon classificationAnimaliaColeopteraCerambycidae

86F1076F-8FE2-5ACA-B25B-D070F85407D4

https://zoobank.org/68605399-2CE3-485B-96B7-2530C1A471B8

Fig. 6

#### Type material examined.

***Holotype***, ♀ (LPSNU), Yen Bai Province, Vietnam, V.2019, local collector leg.

#### Description.

**Figure 6.**
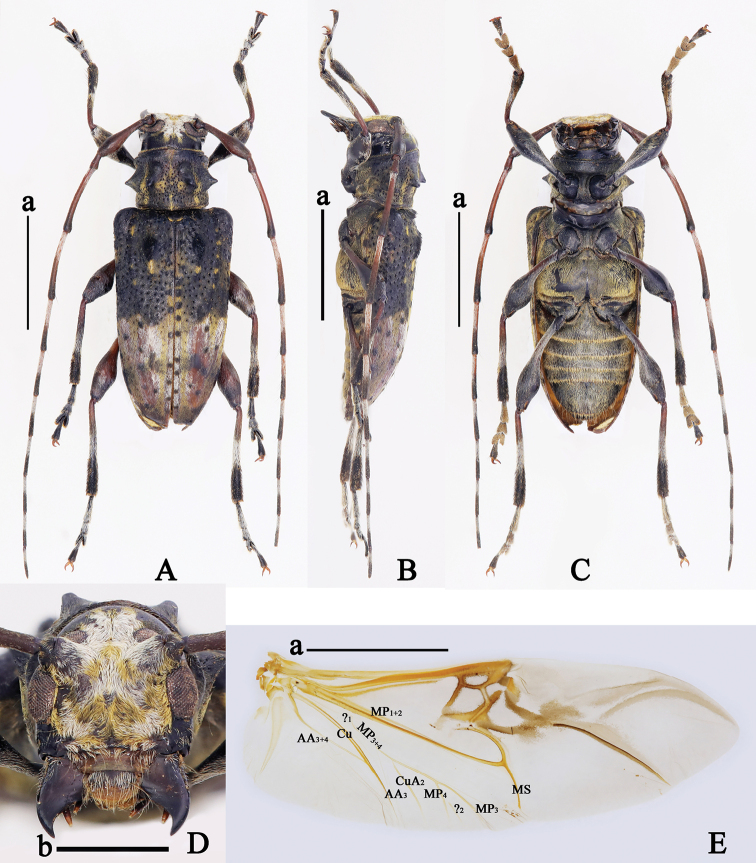
*Trichohoplorananigeralba*, holotype, female **A** habitus, dorsal view **B** habitus, lateral view **C** habitus, ventral view **D** head, frontal view **E** right hind wing, dorsal view. Abbreviations: A: anal, Cu: cubital, MP: medial posterior, MS: medial spur, ?_1_: a vein of uncertain homology (either a crossvein or base of MP_3+4_ vein), ?_2_: an uncertain vein. Scale bars: 5 mm (**a**), 2 mm (**b**).

**Female, holotype.** Body length: 14.0 mm, humeral width: 5.2 mm. Body black, antennal scape (except for outside of apex), pedicel, antennomeres III (except for apex), IV (except for apex), V (basal half), VI (basal half), elytra (apical half), protibiae (basal half), mesofemora (basal half), mesotibiae (basal half), metafemora (most of parts) and metatibiae (basal half) reddish brown; claws yellowish brown (Fig. 6A–C).

Frons (Fig. 6D) densely covered with short yellow and white hairs. Vertex densely covered with short white hairs on middle and short yellow hairs on center (Fig. 6A). Antennae sparsely covered with sub-erect, short, white setae; scape sparsely covered with short, brown setae; pedicel sparsely covered with short, brown setae, with several short, white setae at internal side of apex; antennomere III sparsely covered with short, white setae basally, other parts densely with short, brown setae; antennomeres IV–XI densely covered with short white setae on the basal half and densely with short, brown setae on the apical half; antennomeres III–VII fringed with several long, black setae ventrally; antennomeres VIII–X fringed with one or two long, black setae ventrally; antennae 1.5 times as long as body, length (mm) of each antennomere: scape = 2.7, pedicel= 0.8, III = 3.0, IV= 3.0, V = 2.2, VI = 2.0, VII = 1.7, VIII = 1.6, IX = 1.6, X = 1.5, X I = 1.3; antennomeres III and IV curved inward (Fig. 6A–C). Pronotum (Fig. 6A) covered with three yellow haired bands: two located at sides and starting from near anterior margin to posterior margin, one located in middle and starting from anterior margin to posterior margin; disc with a pair of subtriangular, yellow haired spots located at sides of middle; near anterior of pronotum distinctly expanded outward, pronotum densely punctured (except for apex and base), base of the subuliform tubercles on pronotum expanded forward. Prosternum (sides) and propleuron (venter) sparsely covered with short, yellow hairs (Fig. 6B, C). Scutellum (Fig. 6A) sparsely covered with short black hairs, densely covered with yellow hairs at apex, depressed in middle of apical margin. Elytra (Fig. 6A, B) sparsely covered with short black hairs on the basal half, a short yellow haired band at lateral margins of base, several yellow haired spots arranging into an longitudinal line starting from near posterior humeral angle to basal third, a yellow haired spot located behind the bumps, and several yellow spots along suture from basal fourth to middle; the tubercles at elytral base and near scutellum, and the bumps behind the tubercles densely covered with short, black setae; apical half of each elytron densely covered with short white hairs and four longitudinal yellow haired bands (first band located at lateral margin, second and third bands located in middle and fused at apical half, forth band located near suture); disc 1.9 times as long as wide at base, rounded apically, moderately covered with dense coarse punctures at basal half. Mesosternum, mesepisternum, and mesepimeron sparsely covered with short, yellow hairs; metasternum, metepisternum, and metepimeron densely covered with short, yellow hairs (Fig. 6C). Femora sparsely covered with short white setae and several suberect, long, white setae at external side; tibiae covered with extremely sparse, suberect, long, white setae, sparsely with short, thin, black setae on the basal third, densely with short, white setae in middle, and densely with short, thick, black setae on the apical third; tarsomere I–III (except for venter) densely covered with short white and sparsely suberect, long, white setae dorsally, tarsomere V (except for venter) sparsely covered with short, white setae at basal half and short, black setae on the apical half, with more sparse long black setae at apex (Fig. 6A–C). Abdominal ventrites I–V densely covered with short, yellow hairs, the hairs more dense at apices of ventrites I–IV; apex and sides of ventrite V sparsely covered with long, yellow pubescences (Fig. 6C).

Hind wings (Fig. 6E) with AA_3+4_ vein not bifurcate, AA_4_ vein missed, AA_3_ vein connected with Cu vein near apical 1/3 and not extending to margin; CuA_2_ vein connected with MP_3+4_ vein near basal 1/3 of MP_3+4_ vein and not extending to margin; MP_3+4_ vein bifurcate near apical 1/3, some parts of base of MP_3+4_ vein missed, a short and vague uncertain vein (?_1_, either a crossvein or base of MP_3+4_ vein) connected with base of MP_3+4_ vein; MP_4_ vein, MP_3_ vein and MS vein not extending to margin; a short uncertain vein (?_2_) located between MP_4_ vein and MP_3_ vein, not extending to margin. Abdominal ventrite V raised at apical sides and truncated apically (Fig. 6C).

**Male.** Unknown.

#### Etymology.

The specific epithet of this new species is derived from the Latin words “*niger*” and “*albus*” referring to most of parts of elytral basal half sparsely covered with short, black hairs and most of parts of elytral apical half densely with short, white hairs.

#### Distribution.

Vietnam (Yen Bai).

#### Diagnosis.

This new species can be distinctly distinguished from other species of *Trichohoplorana* by its peculiar elytral pattern (Fig. 6A).

#### Remarks.

When the senior author received the holotype of this new species, the right antennomere XI was missing and the elytral apex was broken. Then, the head, left antennomere XI, and prothorax were separated from the body due to his carelessness, and he correspondingly glued to the body the separated portions with white emulsoid. Consequently, some hairs on the antennae, elytra, metaventrite, and legs were worn so that some characters are unclear.

### ﻿Key to species of *Trichohoplorana*

**Table:** 

1	Elytra with a broad transverse white haired band on the middle	***T.juglandis***
–	Elytra without a broad transverse white haired band on the middle	**2**
2	Elytra not covered with short white or grayish-white hairs	**3**
–	Elytra covered with short white or grayish-white hairs	**4**
3	Tips of the lateral tubercles of the prothorax obtuse, elytral apex slightly obliquely truncated, with the marginal angle obtuse and not distinctly processed, elytral punctuations fine	**T.luteomaculata**
–	Tips of the lateral tubercles of the prothorax pointed, elytral apex distinctly obliquely truncated, with the marginal angle pointed and distinctly stretched, elytral punctuations coarse	**T.shirakii**
4	Most of parts of elytra densely covered with grayish-white hairs	**T.dureli**
–	Most of parts of elytra not densely covered with grayish-white hairs	**5**
5	Elytra with a yellow haired spot located behind bumps, most of parts of elytral apical half densely covered with short white hairs	**T.nigeralba**
–	Elytra without a yellow haired spot located behind bumps, most of parts of elytral apical half not densely covered with short, white hairs	**6**
6	Lateral tubercles of prothorax thick, elytra with a transverse band behind bumps, marginal angles distinctly processed	**T.mutica**
–	Lateral tubercles of prothorax thin, elytra with a transverse band on the middle, rounded apically	**T.tenuipes**

## Supplementary Material

XML Treatment for Trichohoplorana

XML Treatment for Trichohoplorana dureli

XML Treatment for Trichohoplorana juglandis

XML Treatment for Trichohoplorana mutica

XML Treatment for Trichohoplorana tenuipes

XML Treatment for Trichohoplorana shirakii

XML Treatment for Trichohoplorana luteomaculata

XML Treatment for Trichohoplorana nigeralba
